# The Usefulness of SPECT/CT in Sentinel Node Mapping of Early Stage Breast Cancer Patients Showing Negative or Equivocal Findings on Planar Scintigraphy 

**DOI:** 10.22038/aojnmb.2018.10720

**Published:** 2018

**Authors:** Maimoona Siddique, M. Khalid Nawaz, Humayun Bashir

**Affiliations:** 1 Department of Nuclear Medicine, Pakistan Kidney and Liver Institute and Research Centre (PKLI and RC), Lahore, Pakistan; 2 Department of Nuclear Medicine, Shaukat Khanum Memorial Cancer Hospital and Research Centre (SKMCH and RC), Lahore, Pakistan

**Keywords:** Breast cancer, Planar Scintigraphy, Sentinel node, SPECT/CT

## Abstract

**Objective(s):**

This study sought to determine the diagnostic yield of SPECT/CT in localizing axillary sentinel lymph nodes (SLNs) in early breast cancer patients where planar scintigraphy (PS) was equivocal or negative.

**Methods:**

Prospective analysis of early stage breast cancer patients with non-palpable axillary nodes undergoing SLN localization prior to nodal sampling for axillary staging. PS findings were categorized as: Category A: non-visualization of SLN; Category B: unusual uptake location; Category C: equivocal uptake / difficult interpretation. The K-coefficient of Cohen was used to evaluate the correlation between PS and SPECT/CT results. PS and SPECT/CT images were interpreted separately, and SLN identification on each of the modalities was correlated to BMI (Body mass index) and peroperative radio guided results.

**Results:**

Between April 2015 and January 2017, 1028 early breast cancer cases underwent sentinel lymphoscintigraphy. Of total, 134 (13%) patients underwent SPECT/CT in addition to PS. All were females with mean age of 48.15 years (range: 26-82 years). Right sided in 68, left in 64 and 2 with bilateral carcinoma. By TNM classification: 49 (37%) T_1_, 78 (58%) T_2 _and 7 (5%) had DCIS/Paget’s disease.Overall SLNs were detected on both PS and SPECT/CT in 60% cases. Of category A (n=54); 35/54 (64%) SLN localized on SPECT/CT; 32 were level-I; 2 Level-II; 1 Level-III nodes. In 19, SLN was not localized. Of category B (n=18), 5 had prior lumpectomy, SPECT/CT localized tracer uptake to 17 level-I sentinel nodes, 3 level-II and level III / IMC in 9.

Of category C (n=62), 29 had prior lumpectomy. SPECT/CT confirmed SLN in all the cases. Radio-guided surgery confirmed SPECT/CT results. The correlation between the two techniques was low (K=0.34). Where PS was negative; SPECT/CT localized nodes in statistically significant number of cases (=0.01). PS identified SLN uptakes in 80/134 (60%) cases with a mean BMI of 21.6±4.8 kg/m^2^ while SPECT/CT detected ‘‘hot’’ nodes in 115/134 (86%) cases with a mean BMI of 29.6±5.6 kg/m^2^. For overweight/obese patients (n=59) (BMI>25 kg/m^2^), PS failed to identify SLNs in 49 and SPECT/CT failed to do so in 18 (<0.001).

**Conclusion:**

SPECT/CT has diagnostic yield and helps in precise SLN localization where planar imaging is negative or shows unusual site of uptake

## Introduction

Breast cancer is the most common malignancy and the second leading cause of death in females worldwide after lung cancer with considerable geographical and ethnic variations (1). Breast cancer accounts for 23% of all gender and about 42% of malignancies in females over the past 10 years. Pakistan has the highest rate of breast cancer in Asia. Female to male ratio is 100:2. It has been estimated that one out of every 9 females in Pakistan is at risk of developing breast carcinoma at some stage of their lives (2). 

The axillary nodal disease is the most significant prognostic factor for early stage breast cancer patients. Early and accurate nodal staging guides appropriate treatment selection and may show impact on overall prognosis (3-5). Sentinel lymph node biopsy (SLNB) is the most precise method for nodal disease staging in breast cancer, which can diagnose microscopic tumor infiltration to the regional nodes. A fundamental step in the procedure for SLNB is to localize the first tier node of drainage track (6). Axillary nodal disease is discovered in 10–30% of patients with T_1_ (<2 cm) and 45% in those with T_2_ (2-5 cm) breast tumors (7). Routine complete axillary nodal clearance portends higher risk of lymphedema, chronic pain and paresthesias (8).

Planar Scintigraphy (PS) technique is an important step in lymphatic mapping, identifying SLNs in 72-94% of breast cancer patients, but PS mapping is hampered by the absence of anatomical markers, poor resolution and an unpredicted lymphatic drainage pathway (9, 10). A node close to the tracer injection site can also be concealed as the result of intense activity from the injection site (“the shine effect”). Sensitivity and specificity of planar scintigraphy is 94% and 68% respectively (11). 

SPECT/CT (single-photon emission computed tomography) provides corresponding functional and anatomical details, good contrast resolution and has proven superiority over planar imaging under various conditions as it overcomes limitations of PS (12,13). The combination of a SPECT camera and a “low dose CT “ postulates a useful map for the surgeon facilitating surgical exploration and is especially useful in unveiling “hot” nodes not seen on PS, eliminates sites of false-positive uptake sites and accurately pinpoints axillary and extra axillary hot nodes. Attenuation and scatter correction has led to superior SLN identification (11). 

Several studies (14-17) postulated improved anatomical localization of SLNs by SPECT/CT in 98-100% of the examined patient. Ploeg et al. (14) found that SLN was detected by SPECT/CT in 14% cases where PS results were negative. Lerman H. et al. (15) found that PS identified SLNs in 78% patients with a BMI (mean±SD) of 25.2±4 kg/m^2^ and failed to do so in 49 patients (22%) with a BMI of 28±8 kg/m^2^. In 29 of the latter patients (59%), SLNs were mapped on SPECT/CT only. In another study, Lerman et al. (16) reported one case with a tumor positive sentinel node that was visualized by conventional imaging and not depicted by SPECT/CT.

Hybrid SPECT/CT lymphoscintigraphy adds noninvasiveness for detection of SLN particularly in cases with nonvisualization of SLN on PS (17). 

The objective of current study is to explore the added diagnostic yield of hybrid SPECT/CT as compared to PS, mainly in cases where PS results are negative or equivocal. 

As a secondary objective, to enquire whether SPECT/CT has added value to PS particularly in relation to high BMI of breast cancer patients.

## Methods

***Patients’ selection and categorization***

After getting ethical clearance from Institutional Review Board (IRB), 1028 cases of early breast cancer were referred from surgical oncology department of Shaukat Khanum Memorial Cancer Hospital & RC for sentinel lymphoscintigraphy between April 2015 and January 2017. Those with clinically palpable or sonographically detectable metastatic disease were excluded. Of theses, 134 patients (13%) met the criteria for SPECT/CT as described below. Early stage breast cancers T_1_ (Tumor <2 cm), T_2_ (Tumor size 2-5cm) and Ductal carcinoma in situ (DCIS), Paget’s disease with no prior history of chemo/radiotherapy were enrolled in the study prospectively. However, cases with clear visualization of SLN on PS were excluded from analysis. Informed consent was taken from all the included patients.

All cases were further categorized on the basis of PS findings as follows:

***Category A***

Patients with non-visualization of SLN on PS

***Category B***

Unusual location of SLNs

***Category C***

Faint tracer uptakes in axilla those are difficult to interpret, suspicion of skin contamination.

Patients were classified based on weight: those with BMI values of 19.9-24.9 kg/m^2^ were considered to be normal weight; overweight was defined as BMI 25-30 kg/m^2^ and obese as BMI >30 kg/m^2^.

***Preoperative Lymphoscintigraphy Technique***

SLN mapping was done a day before (within 18 hours) of surgery or on the day of surgery. ^99m^Tc labeled human serum albumin colloid particles (^99m^Tc-HSA nano colloids, 20 MBq per injection site) in a volume of 0.2 ml was injected into two intradermal periareolar sites at 6’O clock and 12’O clock positions. PS images were obtained in anterior, anterior oblique and lateral projections on a time based (3 min each view), acquired 45 min post injection by a gamma camera, equipped with a Low Energy High Resolution (LEHR) collimator with energy window set at 140 KeV. A ^57^Co flood source was placed between the patient and camera to define the body contours. SPECT/CT images were acquired immediately after planar images. The SPECT/CT gamma camera consists of dual head variable angle gamma camera equipped with low energy high resolution (LEHR) collimators, 16-slice spiral CT scanner optimized for rapid rotation. SPECT acquisition (matrix 128×128, 60 frames at 25 sec per view) was performed using steps of 6 degree. For CT (130 kV, 20 mA, and B60s kernel), 5-mm slices were created. Both SPECT and CT axial 5-mm slices were generated using an E-soft 2000 application package. The Iterative reconstruction (OSEM 3D) was used for generating SPECT slices. The SPECT data were corrected for attenuation and scatter, and fused with the CT data using software Syngo package. Maximum Intensity Projections (MIP) with a three-dimensional display was generated to localize sentinel nodes in relation to anatomic structures. 

***Image Analysis***

Image analysis was performed prospectively by two experienced nuclear physicians (with experience of 10-15 years in the department of Nuclear Medicine) in consensus reading. SLN localization was interpreted separately on PS and SPECT/CT images. SPECT/CT findings were considered clinically relevant if they clarified SLNs in cases negative on PS, or if SLNs were localized in additional unexpected locations in equivocal PS results. The axillary nodal levels were stratified in relation to their anatomical location as level I, II, III or Internal Mammary Chain (IMC). 

***Correlation of BMI and Lymphoscintigraphic Findings***

The influence of BMI on mapping SLNs by scintigraphy was assessed separately for PS and SPECT/CT images in the study population as well as in the subgroup of 59 overweight and obese breast cancer cases.

***Lymphoscintigraphic Intraoperative Technique***

Pre-operatively, patent blue dye (PBD) was injected (1 ml blue dye injected in four periareolar sites before operation) for SLN visualization. The visible blue stained nodes and those detected by gamma probe were excised. All harvested nodes were fixed in formalin, bisected, embedded in paraffin and histopathological evaluated for presence or absence of metastasis. The surgeon removed all detected SLNs (excluding IMC nodes which are not routinely harvested). If frozen section analysis revealed metastatic SLN, axillary nodal dissection was performed subsequently.

***Statistical Analysis***

All data was entered and assessed by using computer based Statistical Package for Social Sciences (SPSS) version 20. The mean±SD was calculated for quantitative variables like age and BMI. Qualitative variables like gender and presence of SLNs on planar scintigraphy and SPECT/CT were presented in the form of percentages and frequencies. The K coefficient of Cohen’s was used to determine the strength of agreement between PS and SPECT/CT in sentinel detection. Post stratification Chi-square test was applied. P-value≤0.05 was taken as significant. 

Descriptive statistics were used to describe overall patient characteristics as minimum and maximum values. The BMI values (mean±SD) in patients with positive and negative SLNs results on PS or SPECT/CT were calculated and compared. The results of both imaging modalities were compared for all patient categories as well as for overweight (25-30 kg/m^2^) and obese (>30 kg/m^2^).

## Results

***Overall patient’s characteristics***

Of total 1028 early stage breast cancer cases who underwent lymphoscintigraphy, 134 cases fulfilling the inclusion and exclusion criteria were enrolled for SPECT/CT in addition to PS. All were females. Mean age: 48.15 years; age range: 26-82 years. Right sided breast cancer in 68, left in 64 and 2 with bilateral breast carcinoma. By TNM classification: 49 (37%) cases T_1_, 78 (58%) cases T_2 _and 7 (5%) patients had DCIS/ Paget’s disease. 54 cases had prior lumpectomy before presenting for sentinel mapping while the remaining 80 were treatment naive.

***Findings on PS and SPECT/CT***

Overall SLN was detected on both PS and SPECT/CT in 80/134 (60%) cases with equivocal results on PS. PS identified SLN uptakes in 80/134 cases and SPECT/CT localized SLN in 115/134 cases, constituting an identification rate of 59.7% and 85.8% respectively. In 54 cases with negative SLN on PS and constituting 5% of total 1028 cases, SPECT/CT found SLN in 35 cases. So SPECT/CT visualized lymphatic drainage in 115/134 cases and improved visualization rate in 26% cases as compared to PS. The maximal added was seen in those cases that had no SLN detected on PS.

Of category A (n=54) cases, 8 had history of lumpectomy. SPECT/CT localized SLN in 35 cases, 32 level-I nodes, and two Level-II nodes, one Level-III and failed to localize SLN in 19 cases. 

Of category B (n=18) cases, five had prior lumpectomy as well. SPECT/CT localized tracer uptake to 17 level-I sentinel nodes, 3 level-II and 9 level-III / IMC were localized.

**Table 1:** Statistical analysis of results of PS and SPECT/CT

		**Results on SPECT/CT**			**Kappa Value**	**P-value**
		**Present**	**Absent**	**Total**		
**Results on PS**	Present	80	0	80	0.34	0.01
	Absent	35	19	54		
	Total	115	19	134		

**Table 2:** Correlation of BMI and detection of SLN on PS and SPECT/CT images

**BMI (kg/m** ^2^ **)**	**Number of cases**	**PS results**		**SPECT/CT results**		**P-value**
		**Positive**	**Negative**	**Positive**	**Negative**	
≤19.9	36	36		36		
20-24.9	39	34	5	38	1	*0.067
25-29.9	41	7	34	29	12	<0.001
≥30	18	3	15	12	6	0.025
Total	134	80	54	115	19	

* Data for patients with BMI values of less than 19.9 and 20-24.9 (normal weight) were analyzed together.

**Figure 1:**
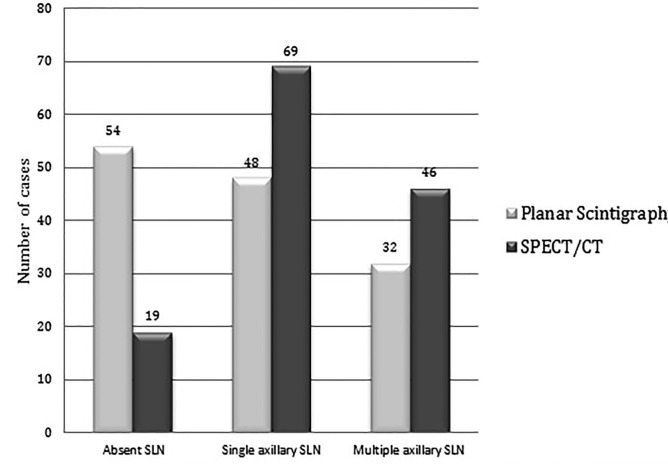
Results of Sentinel Lymphoscintigraphy on PS and SPECT/CT

**Figure 2:**
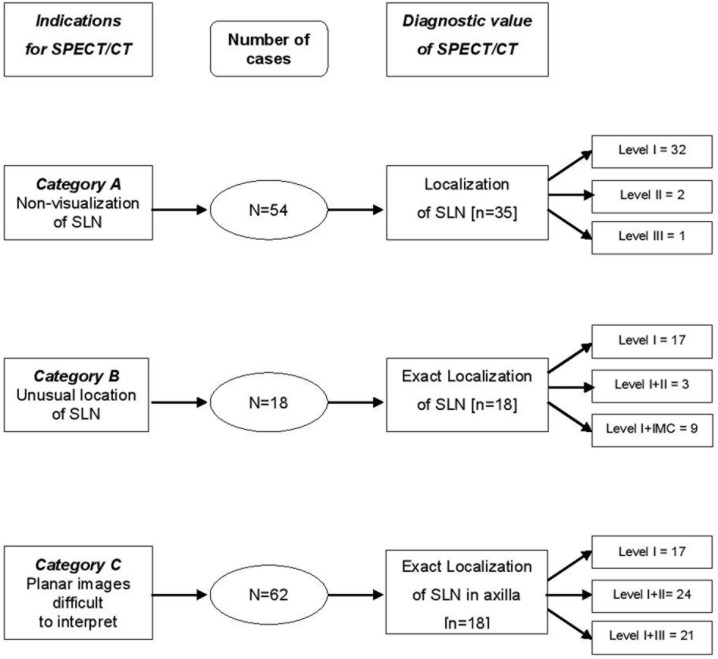
Diagnostic value of SPECT/CT in relation to indications based on PS

SLN: Sentinel Lymph node

IMC: Internal mammary chain nodes

**Figure 3.**
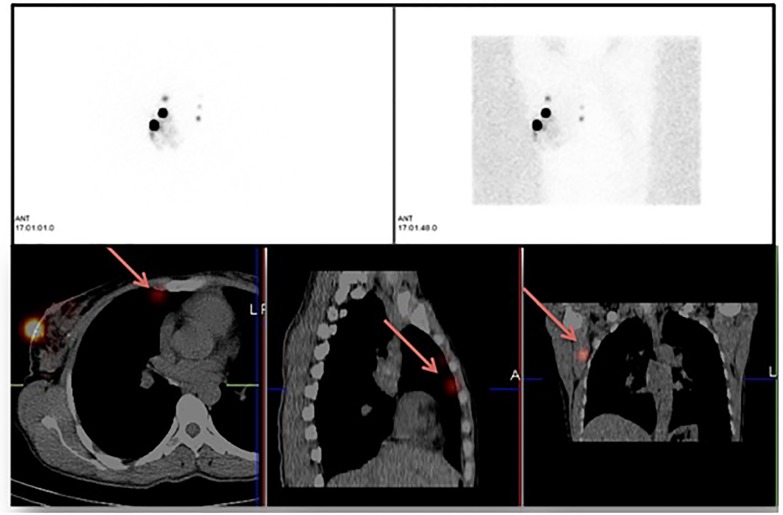
PS images in a female with right breast cancer showed multiple uptakes in medial quadrants of right breast in addition to injection sites. SPECT/CT images localized tracer uptake in right level I axillary SLN and multiple internal mammary chain nodes

**Figure 4:**
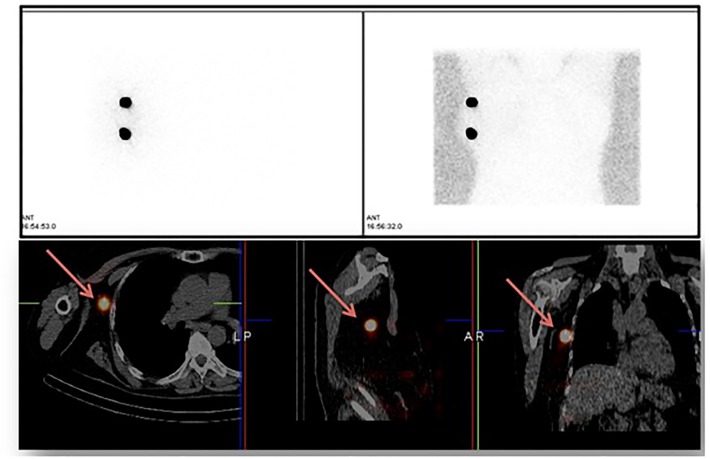
PS images in a female with right breast cancer showed no uptake in right axilla in addition to injection sites. SPECT/CT images localized tracer uptake in right level I axillary SLN

Of category C (n=62) patients, 29 had prior lumpectomy as well. SPECT/CT confirmed SLN in all the cases; no false positive uptake was seen on planar scintigram when compared with SPECT/CT detection of SLN. The radio-guided surgery confirmed the results of SPECT/CT. Results of PS and SPECT/CT are shown in Figure 1 & 2. 

Exemplary PS and SPECT/CT images of breast cancer patients from the current study are shown in Figure 3 & 4.

The correlation between the two techniques was low (K=0.34). Statistically significant difference (=0.01) was noted in SLN mapping between PS and SPECT/CT techniques where lymphoscintigraphic findings were negative. In cases with equivocal findings on PS, SPECT/CT only localized tracer uptake to underlying nodes in addition to identifying more 2^nd^ tier nodes as compared to PS. Its added value was particularly in those cases that were negative on PS results. Statistical correlation is depicted in Table 1. 

BMI range of total study population was 16.8-35.8 kg/m^2^. Of total 134 cases, 59 were overweight and obese. PS identified SLN uptakes in 80/134 (60%) cases with a BMI (mean±SD) of 21.6±4.8 kg/m^2^ while SPECT/CT detected ‘‘hot’’ nodes in 115/134 (86%) cases with BMI of 29.6±6.2 kg/m^2^. For overweight/obese patients (n=59), PS failed to identify SLNs in 49 and SPECT/CT failed to do so in 18.

On subsequent stratification of data based on age (<40 years versus >40 years), tumor size and BMI, it was found that the difference between SLN detection by PS and SLN mapping by SPECT/CT in overweight and obese patients was statistically significant ( value<0.001). The correlation of BMI and lymphoscintigraphic techniques results is summarized in Table 2. This table illustrates the difference in SLN detection by PS and SPECT/CT in patients with different BMI values. The superiority of SPECT/CT appears to be enhanced in overweight and obese patients.

***Operative and Pathological Findings***

115/134 had SLN localization by SPECT-CT lymphoscintigraphy. Intra-operative gamma-probe detected an additional 10 sentinel nodes. In six out of the remaining nine with non-detectable nodes in the axilla, SLNs were identified by PBD staining. No node was identified only by isotopic detection. 

All surgically resected nodes were sent for frozen section histopathology and metastatic nodal disease was found in 29%. These subsequently underwent axillary nodal dissection while the rest with negative frozen section status were spared. 

The three cases with non-detection of SLN by both radioactive and PBD techniques underwent total axillary nodal dissection and histopathological examination revealed metastatic infiltration of nodes in all three.

## Discussion

Breast cancer is the most frequent malignancy affecting females world over. The first lymph node in the lymphatic basin draining the primary tumor is called the sentinel lymph node (SLN). It reflects the histological characteristics of the rest of the nodes in the chain. Metastases to lymph nodes are not a random phenomenon and can be determined by identifying the lymphatic flow from tumor to draining nodal basin (19). 

Our study showed that the addition of SPECT/CT to the acquisition protocol for lymphoscintigraphy might improve the identification of hot nodes in overweight and obese patients. There was statistically significant impact of SPECT/CT for SLN particularly in cases with high BMI>25 kg/m^2^. Such improvement is attributable to both the use of SPECT itself and the improved quality of SPECT images gained by use of CT maps for attenuation correction. CT anatomic landmarks enhance the specificity of SPECT data. SPECT/CT identified hot nodes in 85% of this prospective study, including 35 patients for whom planar imaging failed to detect hot nodes. SPECT/CT detected additional hot nodes missed by planar images alone, including nine IMC nodes and seven level III nodes, which are not routinely explored during surgery. Old age and overweight are reported to be associated with an increased incidence of failed SN identification during surgery. The SLN identification rate increased from 56% for PS to 87% for SPECT/CT (16). Per-operative SLN identification is achieved by injection of a blue dye, which makes the lymphatic vessels and the SLN visible, and by the identification of the radioactive SLN with a hand-held γ-probe (20). In a study of 1,356 patients, Cox et al. found that an increase of one BMI unit decreased the odds of SLNB success by approximately 5% (21). Consequently, patients with high BMI values are more likely to undergo ALND for axillary staging despite the fact that overweight carries a higher risk for postsurgical complications, mainly lymphedema. In current study all the three cases that underwent ALND due to negative SLN detection by both lymphoscintigraphic techniques as well as peroperative gamma probe technique were of high BMI (>25 kg/m^2^). The identification and accurate localization of SLNs in high BMI cases is of major importance, as lymphedema is the most devastating complication of ALND (22).

One hundred and thirty four consecutive patients with diagnosis of intraductal / invasive ductal / lobular breast cancer of early stage T_1_/T_2_ with no clinically palpable axillary lymph nodes were included in this study. The patients underwent both PS and SPECT/CT for sentinel localization one day prior to surgery.

In this study we found that all the patients were females, so gender association could not be evaluated. The mean age was found to be 48.2 years with the greatest number of cases in the age range of 40-60 years. No statistically significant association was seen between detection of SLN and age groups. 

The rate of false-negative planar scintigraphy results for 54, of which 46 cases were overweight and obese patients, was higher than that for the general study population; however, SPECT/CT identified nodes 35/54 cases (64%) and had a statistically higher rate of detection of nodes in overweight patients. 

Older age and high BMI have been reported to be important factors in non-visualization of the sentinel lymph node in several studies, emphasizing the added value of SPECT/CT for SLN localization (23).

Many factors can affect visualization of the sentinel node during lymphoscintigraphy. In infiltrative breast cancers, lymphatic channels become progressively infiltrated with tumor cells and do not allow the passage of radionuclides (24). Cancerous involvement of the lymphatic system may influence the drainage pattern. Completely invaded nodes may lead to unsuccessful axillary node detection due to a lack of ability of tracer uptake in the leading node. Heuser et al. reported 5 cases in which no axillary SN could be detected and consecutive axillary surgery revealed a positive nodal status in 4 of these patients with unsuccessful mapping (25). In our study there were 19/134 (14%) false negative cases on SPECT/CT. The likely cause is related to obliteration of lymphatic channels by tumor cells as suggested by several other authors.

It has previously been reported that age of the patient and tumor size may influence sentinel node detection rates (26). Furthermore, the radiopharmaceutical used, the dose of the pharmaceutical, the particle size of the pharmaceutical, and the injection-to-imaging time may all influence visualization of the sentinel node during Lymphoscintigraphy (27).

In patients with early stage breast cancer, the histological status of SLN is considered representative indicator of the entire lymph node station and strongest predictor of recurrence and survival (5). In this perspective hybrid imaging that combines SPECT and CT has been shown to have an added value compared to conventional PS: the best anatomical design and the best resolution that characterize the SPECT/CT images beyond the limits of PS (28).

The literature reports that the rate of SLN detection using pre-operative PS in breast cancer patients varies from 66% to 95% depending on study population and nuclear physician injection techniques (29-31). In our study, PS and SPECT/CT results were concordant in 115/134 (85%), of which 62 cases with faint/equivocal uptake were clearly identified as positive SLN on SPECT/CT and 19 cases were negative on both PS and SPECT/CT. Disconcordant results were seen in 35/134 (26%) breast cancer cases who were negative on PS but positive SLN detected on SPECT/CT. Moreover, SPECT/CT identified greater number of SLNs than PS. As SPECT/CT showed additional number of SLNs, our study showed statistically significant (=0.01) difference for SLN detection by SPECT/CT (86% for SPECT/CT versus 60% for PS). Although SPECT/CT showed additional number of SLNs as compared to PS but number of nodes seen doesn’t affect the outcome of metastases detection (32).

Despite obvious advantages demonstrated, some authors concluded that SPECT/CT has a limited value in search of SLN, since the technique seems to show similar sensitivity to that of PS in most patients; Moreover, SPECT/CT entails additional cost, more time for the acquisition and exposure to a further dose of radiation (5). However, with SPECT/CT devices using low dose CT, compared to PS alone, added dose of radiation varies from 1.3 to 5 mGy (32). However, the added benefit of exact localization of SLN by SPECT/CT in false negative PS cases outweighs the radiation exposure.

**Limitations of study**

There are few limitations and possible biases in our study. 

As in any other cross-sectional study, we could not exclude the possibility of referral bias that influenced our study. 

The study was conducted over a short period of time. Further studies with larger sample size may be conducted. 

Difference in expertise of injecting physician may also be a cause of high false negative SLN results.

***Limitations of SPECT/CT***

Limitations for SLND with SPECT/CT include extra time and inconvenience for the patient and additional radiation dose. However, the radiation dose is minimal.

According to some studies, the radiation exposure is 1.5 mSv, equivalent to half the dose of two views of mammogram) (11). The quantitative value of Expected Dose (ED) as determined by the reference study (33) is equivalent to the annual natural background radiation of 3 mSv. 

## Conclusion

 In conclusion, the addition of SPECT/CT to the standard imaging protocol for lymphatic mapping and SLN localization in breast cancer patients appears to improve detection of sentinel nodes and their anatomical localization. SPECT/CT has added value particularly in cases with negative, inconclusive or equivocal PS results and provides a better and accurate localization of sentinel nodes in patients presenting drainage outside of the ipsilateral axilla.
